# Complete mitogenome of *Antheraea formosana* Sonan, 1937 (Lepidoptera: Saturniidae): an endemic silkmoth in Taiwan

**DOI:** 10.1080/23802359.2022.2034543

**Published:** 2022-03-06

**Authors:** An-Ping Cheng, Chi-Chun Huang, Yu-Tzu Cheng, Yu-Wei Tseng, Chih-Chiang Wang, I-Ling Lai, Kuo-Hsiang Hung

**Affiliations:** a Graduate Institute of Bioresources, National Pingtung University of Science and Technology, Pingtung, Taiwan; b Taiwan Endemic Species Research Institute, Nantou, Taiwan; c Department of Forestry, Pingtung University of Science and Technology, Pingtung, Taiwan; d Biodiversity Research Center, National Pingtung University of Science and Technology, Pingtung, Taiwan

**Keywords:** *Antheraea formosana*, mitogenome, phylogeny

## Abstract

The complete mitogenome of an endemic silkmoth in Taiwan, *Antheraea formosana*, was determined using Illumina next-generation sequencing. The mitogenome is 15,318 bp in length and consists of 13 protein-coding genes (PCGs), two rRNAs, 22 tRNAs, and one non-coding control region. The overall base composition of the mitogenome showed a high A + T bias, and the A + T content (80.2%) was significantly higher than the G + C content (19.8%). All PCGs use the typical ATN as the initiation codon, with the exception of *cox2*, which begins with GTG, respectively. The complete mitogenome was used to reconstruct a phylogenetic tree, indicating that *A. formosana* is more closely related to *Antheraea assamensis* than other *Antheraea* species, with 93.19% nucleotide similarity.

The tasar silkmoth (*Antheraea* Hübner, 1819) belongs to the Lepidoptera family Saturniidae and is of considerable economic importance worldwide (Kitching et al. 2018). *Antheraea* is the largest genus used for silk production and contains more than 35 described species that are widely distributed throughout Asia (Liu et al. 2008). Currently, the utilization of silkmoths, including *Antheraea pernyi* and *Antheraea assamensis*, for tussah production is greatly prevalent in China, India, and Korea (Peigler 1993; Liu et al. 2010; Li et al. 2017). *Antheraea formosana* Sonan, 1937, a silk-producing Lepidoptera and an endemic species in Taiwan, is a medium to large-sized silkmoth with a wing span of 110–160 mm, which is distributed in low- and middle-altitude mountainous areas (Chowdhury 2004; Wu et al. 2020). Because *A. formosana* behaves as a multivoltine (more than two generations per year), it is a potential alternative candidate for future tussah production in Taiwan. However, there have been no genomic studies on *A. formosana*. In the present study, we report the whole mitogenome sequence of *A. formosana* and reconstruct its phylogeny with other *Antheraea* species.

One *A. formosana* was collected from Taoyuan District, Kaohsiung (23°09′ N, 120°45′ E) in Taiwan. The collection location in this study is not a privately-owned or protected area, and it is not an endangered or protected species in Taiwan. No permits were required for this study. About an abdominal half of a moth was used to extract total genomic DNA by the use of proteinase-K and phenol-chloroform method (Henry et al. 1990). The DNA sample was preserved at the Graduate Institute of Bioresources, National Pingtung University of Science and Technology (Kuo-Hsiang Hung, khhung424@npust.edu.tw), under the voucher number 2021-AF1. The genomic DNA was used for Illumina library preparation, and subsequently, paired-end reads were sequenced using the NovaSeq 6000 platform (Illumina, San Diego, CA, USA). The raw sequences went through a filtering process to obtain the qualified reads by FASTP v.0.20 (Chen et al. 2018), and FLASH v.1.2 was used to merge paired-end reads (Magoč and Salzberg 2011). The complete circular mitogenome of *A. formosana* was assembled de novo using MitoFinder v.1.3 (Allio et al. 2020) from a randomly sampled subset of total genomic reads (23,690,470 reads). The assembled mitogenome was annotated using the MITOS2 web server to predict the location of protein-coding regions/genes, tRNAs, and rRNAs (http://mitos2.bioinf.uni-leipzig.de/index.py) (Donath et al. 2019). The sequence with annotated features was deposited in GenBank (Accession Number OK078922).

We also inferred phylogenetic relationships based on multiple sequence alignments of the other four *Antheraea* species mitogenomes, and *Bombyx mori* was used as an outgroup. The MAFFT online server (Katoh et al. 2019) was used to align the mitochondrial sequences, and a maximum-likelihood tree was constructed based on full mitogenome sequences using MEGA X with 1000 bootstrap replicates (Kumar et al. 2018).

The mitogenome of *A. formosana* was 15,318 bp long. It contains 37 genes, comprising 13 protein-coding genes (PCGs), 22 tRNA genes, and two rRNA genes. The frequencies of adenine, cytosine, guanine, and thymine were 39.3, 12.0, 7.8, and 40.9%, respectively. Therefore, the A + T and G + C contents were 80.2 and 19.8%, respectively. This high A + T bias is similar to that present in other lepidopterans. Twelve PCGs started with a typical ATN codon: four (*nad2*, *cox1*, *atp8*, *nad5*) with ATT, three (*nad3*, *nad6*, *cob*) with ATA, and five (*cox3*, *atp6*, *nad4*, *nad4L*, *nad1*) with ATG. However, *cox2* is associated with GTG. This pattern is similar to that of other *Antheraea* species, except for *cox1*, in which ATT was the start codon. The *cox1* was reported to have CGA as start codons in *Antheraea* species (Singh et al. 2017).

The phylogenetic trees indicated that the *Antheraea* species were separated into two distinct monophyletic clades. *Antheraea formosana* is closely related to *A. assamensis*, with 93.19% nucleotide similarity within the same clade, and other *Antheraea* species clustered in another clade (Figure 1). In conclusion, this research provides useful information for further phylogenetic and evolutionary analyses, as well as enriches the mitogenome database of *Antheraea* species.

**Figure 1.**
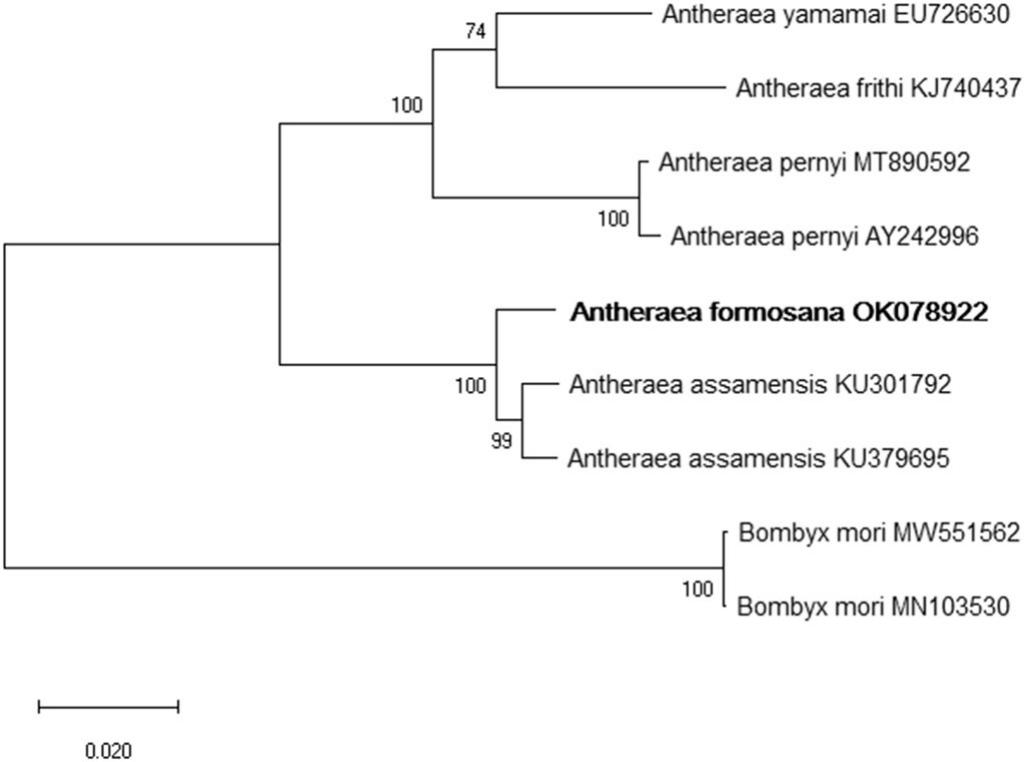
The maximum likelihood (ML) phylogenetic tree indicates the relationship between *Antheraea formosana* and four other *Antheraea* species. *Bombyx mori* was used as an outgroup. GenBank accession numbers of each species are listed in the tree. The numbers on the branch lengths are bootstrap values.

## Data Availability

The genome sequence data are available in GenBank (https://www.ncbi.nlm.nih.gov/) under accession no. OK078922. The associated BioProject, SRA, and Bio-Sample numbers are PRJNA766508, SRR16093533, and SAMN21849811, respectively.
